# Effects of Adaptation of *In vitro* Rumen Culture to Garlic Oil, Nitrate, and Saponin and Their Combinations on Methanogenesis, Fermentation, and Abundances and Diversity of Microbial Populations

**DOI:** 10.3389/fmicb.2015.01434

**Published:** 2015-12-22

**Authors:** Amlan K. Patra, Zhongtang Yu

**Affiliations:** ^1^Department of Animal Sciences, The Ohio State UniversityColumbus, OH, USA; ^2^Department of Animal Nutrition, West Bengal University of Animal and Fishery SciencesKolkata, India

**Keywords:** adaptation, cellulolytic bacteria, garlic oil, saponin, methane, methanogens, nitrate

## Abstract

This study investigated the effects of garlic oil (0.25 g/L), nitrate (5 mM), and quillaja saponin (0.6 g/L), alone and in binary or ternary combinations, on methanogenesis, rumen fermentation, and abundances of select microbial populations using *in vitro* rumen cultures. Potential adaptation to these compounds was also examined by repeated transfers of the cultures on alternate days until day 18. All treatments except saponin alone significantly decreased methanogenesis. Ternary combinations of garlic oil, nitrate, and saponin additively/synergistically suppressed methane production by 65% at day 2 and by 40% at day 18. Feed digestion was not adversely affected by any of the treatments at day 2, but was decreased by the combinations (binary and ternary) of garlic oil with the other inhibitors at days 10 and 18. Saponin, alone or in combinations, and garlic oil alone lowered ammonia concentration at day 2, while nitrate increased ammonia concentration at days 10 and 18. Total volatile fatty acid concentration was decreased by garlic oil alone or garlic oil-saponin combination. Molar proportions of acetate and propionate were affected to different extents by the different treatments. The abundances of methanogens were similar among treatments at day 2; however, garlic oil and its combination with saponin and/or nitrate at day 10 and all treatments except saponin at day 18 significantly decreased the abundances of methanogens. All the inhibitors, either alone or in combinations, did not adversely affect the abundances of total bacteria or *Ruminococcus flavefaciens*. However, at day 18 the abundances of *Fibrobacter succinogenes* and *Ruminococcus albus* were lowered in the presence of garlic oil and saponin, respectively. The results suggest that garlic oil-nitrate-saponin combination (at the doses used in this study) can effectively decreases methanogenesis in the rumen, but its efficacy may decrease while inhibition to feed digestion can increase over time.

## Introduction

Livestock production systems contribute about 12–14.5% to the global anthropogenic greenhouse gas (GHG) emissions in carbon dioxide equivalents (CO_2_-eq; Gerber et al., 2013; Havlík et al., 2014). The direct emissions of methane and nitrous oxide from enteric fermentation and manure management practices (including manure application) contribute 3.5 GtCO_2_-eq GHG to the global emissions annually (Gerber et al., 2013). Enteric methane emission is one of the main sources of GHG from livestock sectors. This contribution will likely continue to increase over the next few decades as the populations of livestock continue to grow to meet the growing demands for meat and milk primarily driven by human population growth and improved standard of living in developing countries (Gerber et al., 2013; Caro et al., 2014; Patra, 2014; Abbasi et al., 2015). Concerns over the contribution from livestock farming to global warming have spurred numerous studies aiming to advance the scientific knowledge on GHG emissions and to develop practical strategies to mitigate GHG emissions from livestock, especially cattle (Patra, 2012; Bellarby et al., 2013; Gerber et al., 2013).

A numbers of methane inhibitors have been repeatedly tried, primarily individually, to decrease enteric methane production (Bozic et al., 2009; Patra, 2012; Hristov et al., 2013; Jayanegara et al., 2015). However, each of them often exerts adverse effects on feed digestion and rumen fermentation when added at high enough doses to achieve effective methane inhibition (Patra and Yu, 2013). In addition, some of these inhibitors are toxic to animals and/or decrease rumen fermentation (Patra, 2012; Hristov et al., 2013). The aforementioned adverse and toxic effects can be overcome at low doses, but unfortunately inhibition to methanogenesis diminishes also. However, combinations of inhibitors with complementary modes of actions may synergistically or additively decrease methane production without any adverse effects on feed digestion or fermentation at low doses (Patra and Yu, 2013). Indeed, in one study using an *in vitro* model of rumen cultures, a binary combination of nitrate (NT) and quillaja saponin (QS) inhibited methanogenesis additively (by 32% at 5 mM NT and 0.6 g/L QS, and by 58% at 10 mM NT and 1.2 g/L QS; Patra and Yu, 2013). Binary NT+QS combination might function additively in decreasing methanogenesis in a multipronged manner: (1) QS functions as an inhibitor to rumen protozoa, lowering hydrogen production by protozoa and decreasing protozoa-associated methanogen abundance (Patra and Saxena, 2009), (2) NT acts as a strong electron sink that outcompetes CO_2_ for electrons, and (3) nitrite, the first intermediate of NT reduction, exerts direct toxicity to methanogens (Bozic et al., 2009; Zhou et al., 2011; Asanuma et al., 2015). However, binary combination of high doses of NT and QS decreased fiber degradability (Patra and Yu, 2013). Garlic oil (GO) is directly inhibitory to rumen methanogens acting through impairment of lipid synthesis (Busquet et al., 2005; Patra and Yu, 2012). It was thus hypothesized that ternary combination of GO+NT+QS could be more effective in decreasing methane production by rumen microbial communities than binary combination of NT+QS. Besides, due to adaptation by rumen microbes, many anti-methanogenic compounds gradually lose efficacy during long-term feeding trials (Patra and Saxena, 2010). The objective of this study was to evaluate the effect of GO, NT, and QS in binary and ternary combinations and adaptation of *in vitro* rumen culture to these compounds on methanogenesis, fermentation, and abundances of select microbial populations.

## Materials and methods

### Experimental design

Garlic oil and QS (from the bark of *Quillaja saponaria* Molina plants) were purchased from Sigma-Aldrich (St. Louis, MO, USA); and sodium nitrate was used as a source of NT. The sapogenin content in the QS product was 24%. The sapogenin content in QS was determined using the gravimetric method (Morris et al., 1958). QS (0.6 g/L), NT (5 mM), and GO (0.25 g/L) were used separately or in binary and ternary combinations, resulting in eight treatments: control (without any methanogenic inhibitor), GO, NT, QS, GO+NT, GO+QS, NT+QS, and GO+NT+QS. These doses were chosen because when used separately they did not affect rumen fermentation or degradability of substrates as shown in previous studies (Busquet et al., 2005; Patra and Yu, 2012, 2015a).

### Preparation of medium, inoculum, and incubation

The inoculum and buffered medium for *in vitro* fermentation was prepared as described previously (Patra and Yu, 2014a). Fresh rumen fluid obtained from two cannulated lactating Jersey cows at around 10 h post morning feeding was used as the inoculum. During the sample collection, the animals were handled following the protocols approved by The Ohio State University Animal Care and Use Committee. The two cows were fed a total mixed ration composed [% dry matter (DM) basis] of corn silage (33%), alfalfa hay (10%), Cargill dairy protein product (Cargill, Inc. Minneapolis, MN; 30%), and a concentrate mixture (27%) twice daily (at 6:00 and 17:00). The rumen fluid collected from each of the two cows was mixed in equal volume and then filtered through three layers of sterile cheesecloth before inoculation. The *in vitro* batch fermentation was carried out in 120-mL serum bottles in triplicate for each treatment (Patra and Yu, 2012, 2013). Because the inocula from two animals were pooled and then incubated in triplicate bottles, the response of the treatments on rumen fermentation and microbial populations may show less variability among the bottle replicates. The use of inocula from each replicate animal would result in true animal variability. The buffered medium for the *in vitro* fermentation was prepared anaerobically (Menke and Steingass, 1988), and 30 ml of the medium and 10 ml of rumen fluid (the inoculum) were dispensed into each serum bottle containing 400 mg of ground feed substrate in an anaerobic chamber. The feed substrate is a mixture of alfalfa hay and a dairy concentrate feed at a 50:50 ratio, with the concentrate feed consisting mainly of ground corn (33.2%), soybean meal (14.2%), AminoPlus® (Ag Processing Inc., Omaha, NE, USA; 15.5%), distillers grains (19.8%), and wheat middlings (11.3%). The headspace of these bottles contained carbon dioxide only. These serum bottles were sealed with a butyl rubber stoppers and incubated at 39°C h in a water bath with intermittent shaking (at 2 h intervals for first 6 h of incubation and then at 8–10 h intervals). The culture adapted to the inhibitors from each bottle (10 ml) was transferred on alternate day to the serum bottles containing same amount of substrate, incubation medium, and methanogenic inhibitors, and the incubation continued until day 18.

### Sampling and chemical analysis

Gas pressure in the culture bottles was measured using a manometer (Traceable®; Fisher Scientific, USA) after 2, 10, and 18 days of incubation to determine total gas production. Subsequently gas sample was collected from each bottle into a glass tube, which was pre-filled with distilled water and sealed with a butyl rubber stopper, by displacement. The gas sample tubes were stored upside down to prevent loss of the gas samples. One milliliter culture was collected from each culture bottle into a microcentrifuge tube for microbial analysis at days 2, 10, and 18. Then, pH values of the *in vitro* cultures were immediately recorded using a pH meter (Fisher Scientific, USA). The remaining content of each culture bottle was filtered through a filter bag (ANKOM Technology, USA) to determine degradability of the feed substrate. The filtrates were sampled into microcentrifuge tubes for analysis for volatile fatty acids (VFA) and ammonia at days 2, 10, and 18. All the samples were stored at −20°C until further analyses.

The concentrations of methane in gas samples were determined using a gas chromatograph (HP 5890 Series, Agilent Technologies, USA) equipped with a thermal conductivity detector and a HP-PLOT Q capillary column coated with porous polymer particles made of divinylbenzene and ethylvinylbenzene (Agilent Technologies Inc, USA). The concentrations of each VFA were also analyzed using a gas chromatograph (HP 5890 series, Agilent Technologies, USA) fitted with a flame ionization detector and a Chromosorb W AW packed glass column (Sigma-Aldrich, USA). The concentrations of ammonia in the fermentation media were determined colorimetrically (Chaney and Marbach, 1962). The degradability of DM and neutral detergent fiber (NDF) of the substrate was determined gravimetrically (Blümmel et al., 1997) at days 2, 10, and 18.

### Extraction of DNA, qPCR, and denaturing gradient gel electrophoresis (DGGE)

Metagenomic DNA was extracted from each culture sample following the procedure described by Yu and Morrison (2004a). The DNA quality was evaluated using agarose gel (1%) electrophoresis, and DNA yield was quantified using the Quant-iTdsDNA Broad Range Assay kit (Invitrogen Corporation, Carlsbad, CA, USA) on a Stratagene Mx3000p machine (La Jolla, CA, USA). The DNA samples were stored at −20°C until analyses.

The abundances of total archaea, total protozoa, and select bacterial species were quantified using SYBR Green-based quantitative real time-PCR (qPCR) using a Stratagene Mx3000p machine following the procedure described earlier (Patra and Yu, 2014a). Briefly, sample-derived qPCR standards were prepared using the respective specific PCR primer sets (e.g., Patra and Yu, 2014a) and a composite DNA sample that was prepared by pooling an equal amount of all the metagenomic DNA samples (Yu et al., 2005; Patra and Yu, 2014a). The standards were then purified using a PCR Purification kit (Qiagen, USA) and quantified. For each of the standards, 16S rRNA (*rrs*) gene copy numbers were calculated based on the length of the PCR products and the mass concentrations (Yu et al., 2005). Tenfold serial dilutions were prepared in Tris-EDTA buffer prior to qPCR assays. To minimize variations, the qPCR assay for each species or group of the microbes was performed in triplicate for both the standards and the metagenomic DNA samples using the same master mix and the same PCR plate. The absolute abundances were expressed as *rrs* gene copies/mL of culture samples.

The microbial community in each of the cultures was profiled using domain-specific PCR and DGGE (Yu and Morrison, 2004b; Yu et al., 2008). Briefly, the V3 region of the 16S rRNA gene of bacteria and archaea was amplified using bacteria- and archaea-specific primers with a 40 bp GC clamp attached at the 5′ end of the forward primers (Patra and Yu, 2013). After confirmation by agarose gel (1.2%) electrophoresis, all the PCR products were resolved using polyacrylamide gels (8%) containing a 40–60% linear denaturing gradient (Patra and Yu, 2013). Following staining with SYBR green I (Molecular Probe, Oregon, USA), the banding patterns were captured using a FlourChem Imaging System (Alpha Innotech Corporation, San Leandro, CA, USA) and then analyzed using the BioNumerics software (Applied Maths, Inc., Texas, USA).

### Statistical analysis

The data were analyzed using the PROC MIXED procedure of SAS (version 8, SAS Inst. Inc., Cary, NC, 2001) with a model containing treatment and day as the repeated measures with serum bottle as subject, and interaction between treatment and day as main effects. Compound symmetry was selected as covariance structure according to the best fit using the Akaike information criterion. Significance was declared at *P* < 0.05, whereas 0.05 < *P* < 0.10 were considered as a trend. When the interaction between treatment and day was significant, the SLICE option in the LSMEANS statement was used to determine differences among the treatments at each time point. The abundances (*rrs* gene copies/mL culture) of ruminal microorganisms quantified were log-transformed before statistical analyses to improve normality. Principal component analyses (PCA) of DGGE profiles of bacteria and archaea were performed using SAS based on the peak heights and migration of the bands after logarithmic transformation to account for normal distribution of the data (Patra and Yu, 2012; Patra et al., 2012). Graphically, the PCA plot visualizes the relative similarity of community composition as indicated by the distance among treatments.

## Results

### Effects of garlic oil, nitrate, saponin, and their combinations on total gas and methane production, and feed degradability

Total gas and methane production, feed degradability, ammonia concentration were affected by treatment, day, and treatment × day interaction (Table 1). At day 2, total gas production was the lowest for the combination of GO+NT+QS followed by the combination of NT+QS, and GO and NT alone, whereas total gas production was similar among control, QS, GO+QS, and GO+NT. At day 10, only the combinations of GO (both binary and ternary) with NT and QS significantly decreased gas production. At day 18, total gas production was lower for GO alone and its combinations (both binary and ternary) with NT and QS than for NT and NT+QS. Inclusion of QS alone in the culture had no effect on gas production on any days. All the treatments except QS alone decreased methane production until day 18 compared with the control. Generally, combinations of additives caused greater methane inhibition compared with the additives included in the culture separately. Combination of GO+NT+QS additively decreased methane production compared with these compounds alone or their binary combinations at day 2. This effect generally persisted until day 18. The ternary combination of additives decreased methane production by 65, 61, and 39% at day 2, 10, and 18, respectively, compared with the control. At day 2, degradability of DM or NDF was similar among the treatments. However, degradability of DM and NDF was substantially decreased by GO alone and its combinations (both binary and ternary) with NT and QS at day 10 and 18. Other treatments had no effect on the degradability of feed until day 18. The degradability of DM was lower by about 7% units at day 10–18 for the ternary combination than for the control. At day 2, concentrations of ammonia were lower for QS alone and its combinations (both binary and ternary) with the other compounds and GO alone than for the control. In contrast, NT alone and its combinations (both binary and ternary) with GO and QS increased ammonia concentration compared with the control on both days 10 and 18. Compared with the control, the ternary combination lowered concentration of ammonia by 28% at day 2, while ammonia concentration increased by 28 and 14% at day 10 and 18, respectively, for the ternary combination. The values of pH were not affected (*P* = 0.085) by either the treatments or treatment × day interaction.

**Table 1:** **Effects of garlic oil, nitrate, quillaja saponin, their combinations, and length of adaptation period on total gas and methane production (ml), degradability (%) of feeds and ammonia concentration (mM) in ***in vitro*** rumen mixed culture**.

	**Treatment (T)**								**SEM**	**Effects**		
	**Control**	**GO**	**NT**	**QS**	**GO+NT**	**GO+QS**	**NT+QS**	**GO+NT+QS**		**T**	**D**	**T × D**
**TOTAL GAS**												
Day 2	83.4^c^	73.9^b^	75.2^b^	80.2^c^	82.9^c^	79.6^c^	74.4^b^	67.1^a^	0.884	<0.001	<0.001	<0.001
Day 10	77.3^cd^	73.5^c^	73.9^c^	81.3^d^	60.1^a^	65.8^b^	73.0^c^	61.7^ab^				
Day 18	86.6^d^	72.4^b^	79.8^c^	84.6^d^	68.4^a^	73.0^b^	76.6^c^	66.6^a^				
**METHANE**												
Day 2	29.1^d^	20.6^bc^	22.0^bc^	24.9^cd^	24.2^c^	21.1^bc^	18.4^b^	10.3^a^	0.665	<0.001	<0.001	<0.001
Day 10	19.3^cd^	16.3^bc^	14.6^b^	20.5^d^	8.0^a^	8.7^a^	15.1^b^	7.6^a^				
Day 18	21.4^d^	14.8^b^	14.8^b^	21.5^d^	14.4^ab^	18.1^c^	15.1^b^	13.0^a^				
**DMD**												
Day 2	73.6^ab^	71.6^a^	74.9^b^	75.2^b^	73.1^ab^	74.2^b^	74.6^b^	71.5^a^	0.871	<0.001	<0.001	<0.001
Day 10	72.3^c^	70.1^bc^	73.7^c^	72.8^c^	66.6^ab^	64.7^a^	71.4^bc^	63.4^a^				
Day 18	75.0^cd^	66.7^ab^	76.0^d^	73.0^c^	67.9^b^	65.4^a^	74.9^cd^	65.6^ab^				
**NDFD**												
Day 2	63.0^ab^	55.7^a^	63.2^ab^	65.2^b^	60.0^ab^	62.2^ab^	63.3^ab^	56.5^ab^	1.86	<0.001	<0.001	<0.001
Day 10	63.5^cd^	60.7^bcd^	66.2^d^	64.5^cd^	55.5^abc^	53.2^ab^	63.2^cd^	49.6^a^				
Day 18	59.7^b^	37.3^a^	60.0^b^	57.2^b^	39.4^a^	35.0^a^	58.2^b^	36.5^a^				
**AMMONIA**												
Day 2	26.3^bc^	21.1^a^	27.4^c^	17.9^a^	22.5^abc^	18.8^a^	21.6^a^	18.8^a^	0.965	<0.001	<0.001	<0.001
Day 10	20.3^b^	22.4^bc^	31.9^d^	23.8^bc^	25.5^c^	14.3^a^	25.5^c^	26.0^c^				
Day 18	19.5^ab^	19.7^ab^	28.8^d^	16.6^a^	22.1^bc^	17.2^a^	26.2^cd^	22.3^bc^				
**pH**												
Day 2	6.65	6.60	6.68	6.50	6.51	6.49	6.51	6.47	0.085	0.10	0.001	0.25
Day 10	6.62	6.34	6.69	6.52	6.76	6.61	6.60	6.75				
Day 18	6.65	6.74	6.74	6.59	6.83	6.73	6.69	6.83				

*GO, garlic oil; NT, nitrate; QS, quillaja saponin; T, treatment; D, day; SEM, standard error of mean; DMD, dry matter degradability; NDFD, neutral detergent fiber degradability. Means followed by different superscript letters (a–d) in a row differ significantly (P < 0.05) among the treatments*.

### Effects of garlic oil, nitrate, saponin and their combinations on fermentation characteristics of ruminal cultures

Total VFA concentration and molar proportion of individual VFAs were affected (*P* < 0.001) by treatment and by treatment × day interaction (Table 2). Total VFA concentrations were similar among the treatments at day 2, but were lower for GO, GO+QS, and GO+NT+QS than for that of the control at day 18. At day 10, GO+QS decreased total VFA concentrations compared with the control. Other treatments had little effect on total VFA concentrations. Total VFA concentration decreased in GO+NT+QS combination by 15% at day 18 compared that in the control. At days 2 and 10, acetate molar proportion was lower for GO+QS, but was higher for NT and NT+QS than for the control. At day 18, molar proportion of acetate was similar among all treatments except for GO+NT that increased acetate percent. Molar proportion of propionate increased in the presence of QS compared to that of the control at day 2, but it decreased in the presence of NT at day 10. At day 18, GO alone or in combinations (both binary and ternary) with NT and QS, and the NT+QS combination reduced molar percent of propionate. At day 2, molar proportion of butyrate decreased in the presence of NT and NT+QS in the culture media. At day 10, QS alone, GO+NT, GO+QS, and GO+NT+QS increased molar percent of butyrate. At day 18, GO alone and in combinations (both binary and ternary) with NT and QS increased molar proportion of butyrate. Compared with the control, molar proportion of isobutyrate was not affected by any of the treatments at day 2, but it was increased by QS alone and its combination (both binary and ternary) at day 10. The molar proportion of iso-butyrate was decreased by GO+QS and GO+NT+QS at day 18 compared to that of the control. Compared to the control, proportion of valerate was lower for NT+QS at day 2 and for QS alone and NT+QS at day 10, but it increased by GO+QS and GO+NT at day 10 and by GO alone and GO+QS at day 18. Proportion of iso-valerate was lowered by QS at day 2, but it was lowered by GO at days 10 and 18. At day 2, acetate to propionate ratio (A:P) was lower (*P* < 0.05) for QS and its combination with NT and GO (i.e., GO+QS, NT+QS, and GO+NT+QS) compared with the control. However, at day 10, this ratio was only lower for the combination of GO+QS, but was higher for NT and its combination with GO and QS (i.e., GO+NT, NT+QS, and GO+NT+QS) than for the control. At day 18, A:P was higher for all treatments than for the control except for QS alone that did not influence this ratio.

**Table 2:** **Effects of garlic oil, nitrate, quillaja saponin, their combinations, and length of adaptation period on total volatile fatty acid (VFA) concentration (mM) and percent of individual VFA in ***in vitro*** rumen culture**.

	**Treatment (T)**								**SEM**	**Effects**		
	**Control**	**GO**	**NT**	**QS**	**GO+NT**	**GO+QS**	**NT+QS**	**GO+NT+QS**		**T**	**D**	**T × D**
**TOTAL VFA**												
Day 2	108.5	103.2	105.9	106.3	110.5	111.2	106.2	105.4	2.01	<0.001	<0.001	<0.001
Day 10	77.7^bcd^	76.1^bc^	81.7^cd^	84.0^d^	71.1^ab^	67.8^a^	82.3^cd^	70.5^ab^				
Day 18	86.1^b^	74.2^a^	82.0^ab^	85.1^b^	81.9^ab^	75.1^a^	86.8^b^	73.2^a^				
**ACETATE (A)**												
Day 2	58.6^bc^	57.7^ab^	61.9^d^	58.9^bc^	61.3^cd^	55.6^a^	61.8^d^	60.1^bcd^	0.780	<0.001	0.008	0.001
Day 10	58.5^b^	58.5^b^	62.3^c^	58.5^b^	60.1^bc^	54.6^a^	61.6^c^	60.4^bc^				
Day 18	57.3^a^	61.5^ab^	62.2^ab^	58.8^ab^	63.3^b^	59.2^ab^	58.9^ab^	62.4^ab^				
**PROPIONATE (P)**												
Day 2	20.4^a^	21.3^ab^	20.8^a^	23.6^c^	21.2^ab^	25.5^c^	23.4^bc^	24.6^c^	0.443	<0.001	<0.001	<0.001
Day 10	26.0^bc^	27.6^c^	22.1^a^	23.8^ab^	21.8^a^	27.8^c^	22.1^a^	21.8^a^				
Day 18	25.6^d^	21.4^ab^	24.2^cd^	25.7^d^	19.8^a^	21.6^b^	23.3^c^	20.7^ab^				
**ISO-BUTYRATE**												
Day 2	1.82^ab^	1.61^ab^	1.67^ab^	1.47^a^	1.86^ab^	1.97^b^	1.47^a^	1.59^ab^	0.120	<0.001	<0.001	<0.001
Day 10	1.48^a^	1.90^ab^	2.08^ab^	2.24^b^	3.52^bc^	3.11^cd^	2.53^d^	3.40^d^				
Day 18	2.20^b^	1.74^ab^	1.68^ab^	1.59^ab^	1.92^ab^	1.48^a^	1.87^ab^	1.49^a^				
**BUTYRATE**												
Day 2	12.5^d^	13.1^d^	9.4^bc^	10.3^c^	9.9^c^	10.5^c^	8.24^a^	8.50^ab^	0.230	<0.001	<0.001	<0.001
Day 10	7.78^a^	7.32^a^	7.97^ab^	9.12^cd^	9.20^cd^	9.41^d^	8.08^ab^	9.65^d^				
Day 18	8.54^a^	11.8^c^	8.94^a^	8.34^a^	10.0^b^	12.6^c^	8.80^a^	10.8^b^				
**ISO-VALERATE**												
Day 2	4.01^c^	3.60^bc^	3.76^bc^	3.22^ab^	3.34^ab^	3.64^bc^	2.92^a^	2.90^a^	0.177	<0.001	<0.001	<0.001
Day 10	3.49^cd^	2.17^ab^	3.14^bc^	4.34^d^	2.02^a^	1.71^a^	3.60^cd^	1.90^a^				
Day 18	4.09^c^	2.84^ab^	3.16^b^	3.12^b^	2.48^ab^	2.22^a^	4.66^c^	2.22^a^				
**VALERATE**												
Day 2	2.72^b^	2.74^b^	2.51^ab^	2.49^ab^	2.38^ab^	2.53^ab^	2.18^a^	2.31^ab^	0.098	<0.001	<0.001	<0.001
Day 10	2.76^b^	2.44^ab^	2.42^ab^	2.00^a^	3.39^c^	3.41^c^	2.11^a^	2.88^bc^				
Day 18	2.27^a^	3.12^b^	2.08^a^	2.39^a^	2.42^a^	2.91^b^	2.45^a^	2.34^a^				
**A–P Ratio**												
Day 2	2.88^cd^	2.71^bc^	2.98^d^	2.50^ab^	2.88^cd^	2.19^a^	2.64^bc^	2.45^ab^	0.063	<0.001	<0.001	<0.001
Day 10	2.25^b^	2.1^ab^	2.83^d^	2.46^bc^	2.76^d^	1.98^a^	2.80^d^	2.77^d^				
Day 18	2.25^a^	2.88^cd^	2.57^b^	2.29^a^	3.19^e^	2.74^bc^	2.53^b^	3.01^de^				

*GO, garlic oil; NT, nitrate; QS, quillaja saponin; T, treatment; D, day; SEM, standard error of mean. Means followed by different superscript letters (a–e) in a row differ significantly (P < 0.05) among the treatments*.

### Effects of garlic oil, nitrate, saponin, and their combinations on abundance of archaea, protozoa, and select cellulolytic bacteria

Abundances of methanogens, cellulolytic bacteria, and protozoa were affected by treatment × day interaction (Table 3). On average of the same sampling days, total bacterial abundance was higher for QS, GO+NT, NT+QS, and GO+NT+QS than for the control. Abundance of *F. succinogenes* was higher for the combinations containing QS than for GO alone, but was similar to that of the control at day 2. The GO alone and all the combinations containing GO appeared to be inhibitory to *F. succinogenes* at day 10, with more inhibiting effect at day 18. The GO generally caused a growth inhibition of *F. succinogenes* by 1.0 and 2.0 log units at day 10 and 18, respectively, compared with the control. The population sizes of *R. flavefaciens* were similar among all treatments at days 2, 10, and 18 except for a larger population of *R. flavefaciens* in GO than in NT+QS at day 18. *R. albus* was not affected by any of the treatments at day 2. The abundance of *R. albus* was increased by GO and GO+NT, but decreased by GO+NT+QS compared to that of the control at day 10. At day 18, QS alone or all the combinations containing QS decreased the abundance of *R*. *albus*. Compared to the control, the abundance of *R. albus* was lower for the ternary combination by 2 and 2.5 log units at day 10 and 18, respectively. Archaeal populations were not affected by any of the treatments at day 2, whereas GO individually or in combinations with NT and/or QS inhibited the growth of archaea on day 10. At day 18, all the treatments except QS alone decreased the abundances of archaea. Compared with the control, the ternary combination inhibited the growth of archaea by 1.8 log units at day 10, which was more profound (by 3.2 log units) at day 18. Protozoal population was lower for QS, NT+QS, GO+NT, and GO+QS than that of the control at day 2; however, none of the treatments affected the protozoal abundance at day 10 or 18.

**Table 3:** **Effects of garlic oil, nitrate, quillaja saponin, their combinations, and length of adaptation period on abundances (log_**10**_16S rRNA gene copies/ml) of select microbial populations in ***in vitro*** rumen culture**.

	**Treatment (T)**								**SEM**	**Effects**		
	**Control**	**GO**	**NT**	**QS**	**GO+NT**	**GO+QS**	**NT+QS**	**GO+NT+QS**		**T**	**D**	**T × D**
**TOTAL BACTERIA**												
Day 2	11.73^a^	11.86^abc^	11.77^ab^	12.16^bc^	12.11^abc^	11.73^a^	12.19^c^	12.10^abc^	0.080	<0.001	<0.001	0.14
Day 10	11.70	11.79	11.63	11.77	11.94	11.68	11.81	11.81				
Day 18	11.73	11.76	11.78	11.78	11.78	11.70	12.02	11.82				
Mean	11.72^a^	11.80^ab^	11.73^a^	11.90^bc^	11.94^c^	11.71^a^	12.0^c^	11.91^bc^				
***F. SUCCINOGENES***												
Day 2	7.24^ab^	6.42^a^	7.20^ab^	7.18^ab^	7.30^ab^	7.91^b^	7.70^b^	7.77^b^	0.239	<0.001	<0.001	<0.001
Day 10	8.62^b^	7.62^ab^	8.54^b^	8.62^b^	7.15^a^	7.30^a^	8.61^b^	7.78^ab^				
Day 18	9.57^b^	6.98^a^	9.37^b^	9.22^b^	6.54^a^	6.30^a^	9.56^b^	6.77^a^				
***R. FLAVEFACIENS***												
Day 2	8.80	9.25	9.00	9.17	9.56	8.85	9.24	9.29	0.197	<0.001	<0.001	0.001
Day 10	9.06	9.33	9.11	8.80	9.46	9.03	8.89	9.31				
Day 18	8.27^ab^	9.30^b^	8.35^ab^	7.38^a^	8.47^ab^	8.82^ab^	7.67^a^	8.75^ab^				
***R. ALBUS***												
Day 2	6.27	6.88	6.59	6.78	7.20	6.57	7.07	6.78	0.214	<0.001	<0.001	<0.001
Day 10	6.00^bc^	7.16^d^	6.74^cd^	5.33^b^	6.83^d^	4.82^ab^	5.69^bc^	4.00^a^				
Day 18	7.72^b^	8.01^b^	7.70^b^	5.48^a^	7.95^b^	5.89^a^	5.38^a^	5.10^a^				
**ARCHAEA**												
Day 2	7.19	7.10	7.35	7.37	7.43	7.51	7.28	7.16	0.162	<0.001	<0.001	<0.001
Day 10	7.88^d^	6.86^abc^	7.11^bcd^	7.80^cd^	6.03^a^	6.30^ab^	7.09^bcd^	6.10^ab^				
Day 18	9.44^e^	6.74^b^	8.18^cd^	8.91^de^	5.91^a^	6.23^ab^	7.70^c^	6.20^ab^				
**PROTOZOA**												
Day 2	7.25^c^	6.93^bc^	7.02^bc^	6.49^ab^	6.39^a^	6.19^a^	6.51^ab^	7.17^c^	0.185	0.24	<0.001	0.006
Day 10	5.58	5.11	5.39	5.19	5.22	5.09	4.92	5.28				
Day 18	4.79	5.24	5.29	5.34	5.33	5.25	5.48	5.12				

*GO, garlic oil; NT, nitrate; QS, quillaja saponin; T, treatment; D, day; SEM, standard error of mean. Means followed by different superscript letters (a–e) in a row differ significantly (P < 0.05) among the treatments*.

### Effects of garlic oil, nitrate, saponin, and their combinations on communities of bacteria and archaea

**Figure 1:**
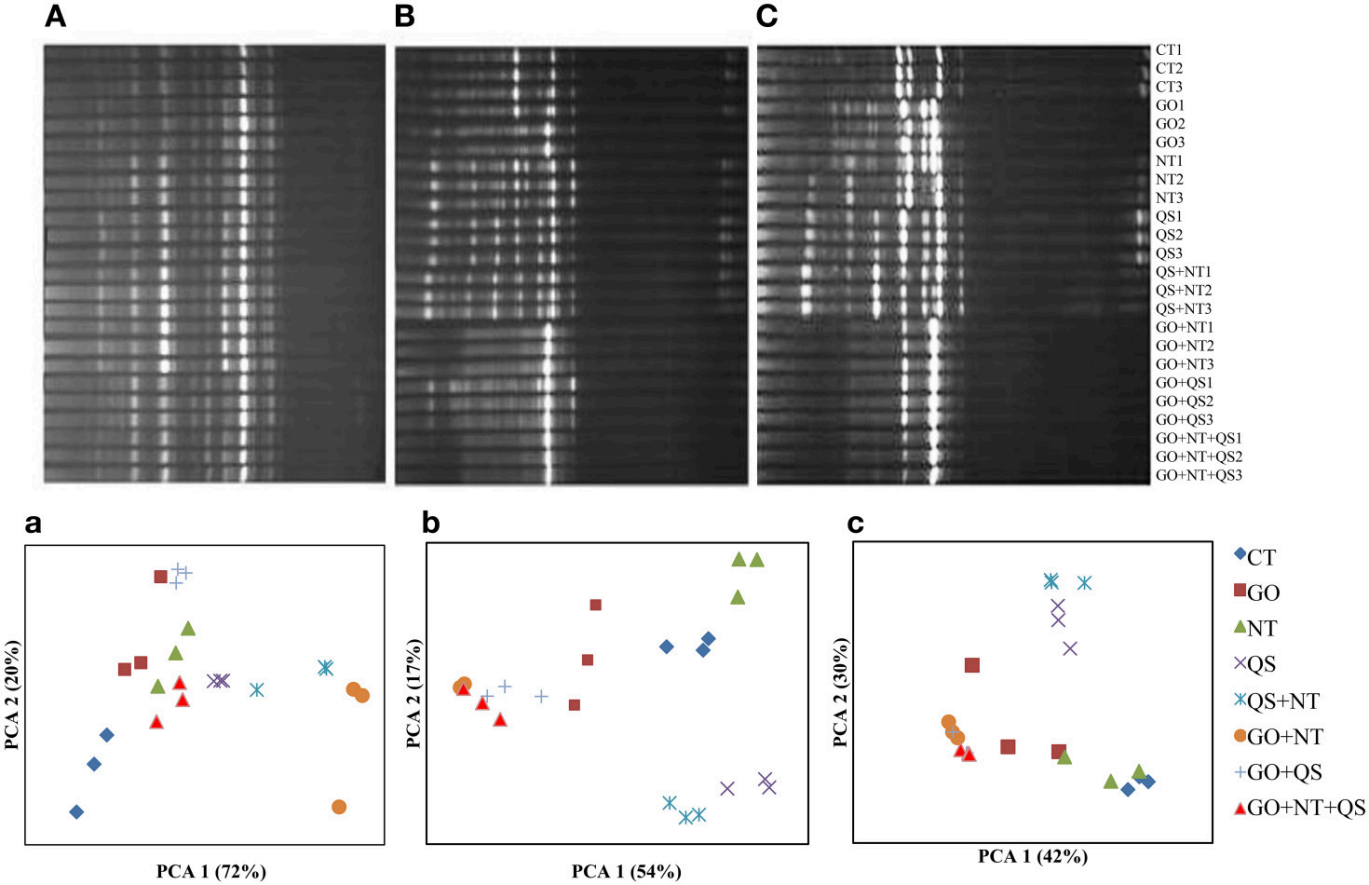
**DGGE profiles (A–C) and PCA plots (a–c) of archaeal communities at day 2 (A and a), day 10 (B and b), and day 18 (C and c)**. CT, GO, NT, and QS stand for the control, garlic oil, nitrate, and quillaja saponin, respectively. All the treatments were replicated in triplicates.

The DGGE profile of archaea showed that the inhibitors changed their community structures drastically (Figure 1). Many of the DGGE bands disappeared at days 10 and 18, especially at day 18 in GO+QS, GO+NT, and GO+QS+M, while a few bands were intense. In the PCA plots, PCA 1 and PC 2 explained variances of 72 and 20%, respectively, at day 2, 54 and 17%, respectively, at day 10, and 42 and 30%, respectively, at day 18. Distinct communities were much evident at days 10 and 18. At day 10 and 18, the archaeal populations in QS and QS+NT were clustered together, and GO in combination with QS and N produced similar community structures.

**Figure 2:**
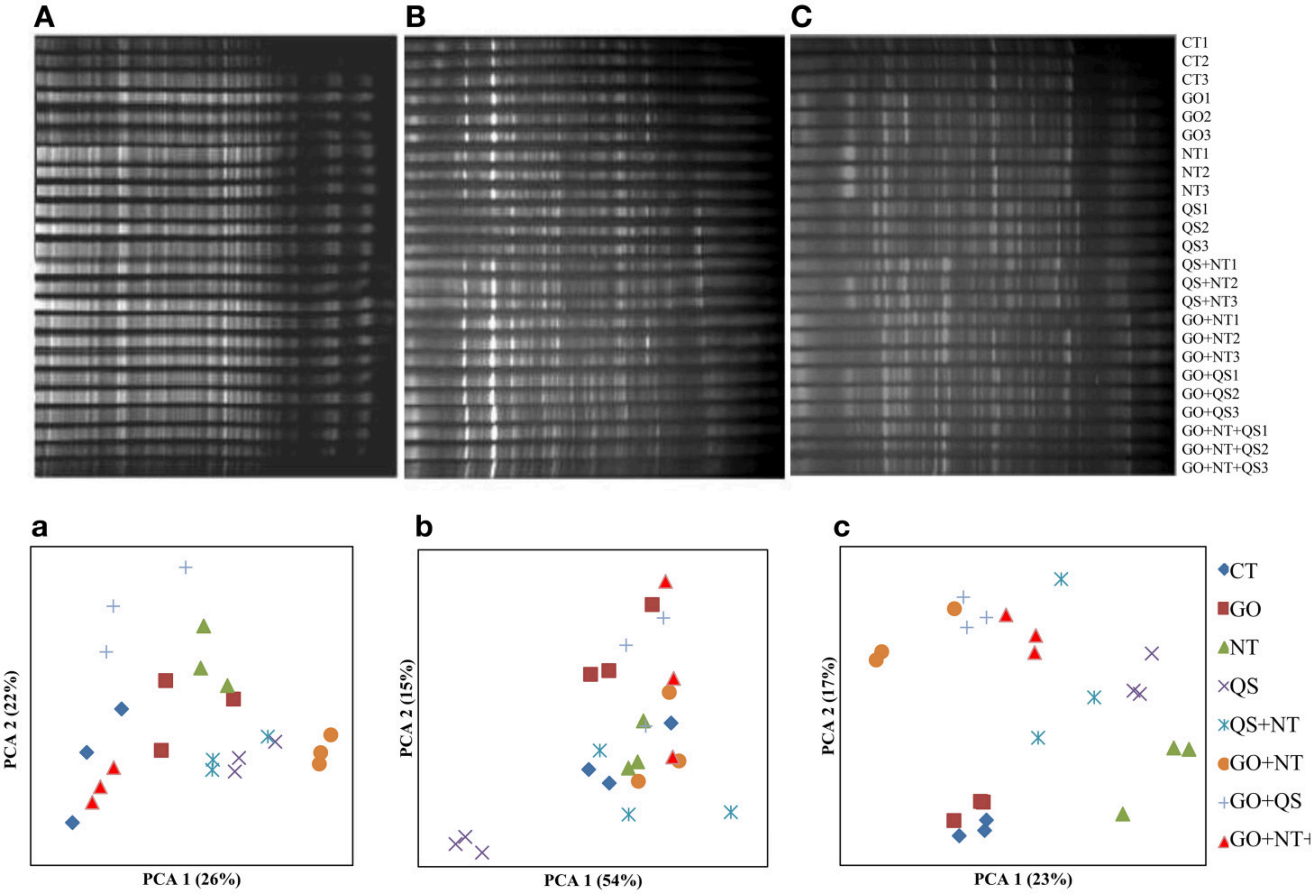
**DGGE profile (A–C) and PCA plots (a–c) of bacterial communities at day 2 (A and a), day 10 (B and b), and day 18 (C and c)**. CT, GO, NT, and QS stand for the control, garlic oil, nitrate, and quillaja saponin, respectively. All the treatments were replicated in triplicates.

The bacterial community structure though changed due to addition of the inhibitors in the cultures, it was less distinct compared with that of the archaeal communities (Figure 2). The PCA 1 and PC 2 explained variances of 26 and 22%, respectively, at day 2, 54 and 15%, respectively, at day 10, and 23 and 17%, respectively, at day 18. At day 2, QS yielded a distinct bacterial community separated from other communities. At day 18, the bacterial communities of the control and GO clustered together.

## Discussion

Practical dietary mitigation of methane emission from ruminant animals faces two challenges: (i) adverse effect on feed digestion and fermentation at effective doses and (ii) gradual loss of efficacy over long-term use. In a recent *in vitro* study with 24 h incubation, we demonstrated that combination of NT (5 mM), GO (0.25 g/L), and QS (0.6 g/L) effectively decreased methane production by rumen microbiome without significantly decreasing feed substrate degradation or fermentation (Patra and Yu, 2015a). In the present study, we aimed to determine if rumen microbiome can adapt to these inhibitors during long-term exposure. All the compounds, either alone or in their combination, decreased methane production significantly over the course of the experiment except QS that decreased methane by 14.4% at day 2 but lost the ability to lower methane production at day 10 and 18. These results suggest that rumen microbiome may be able to overcome inhibition by QS through adaptation. The anti-methanogenic action of GO may be mediated through interaction with the cell membrane and cellular enzymes. Archaea are thought to be sensitive to GO as some of its organosulfur compounds may inhibit some of the SH-containing enzymes essential to metabolic activities, especially those involved in synthesis of specific isoprenoid side chains in archaeal lipids that are absent in rumen bacteria (Calsamiglia et al., 2007; Patra and Yu, 2012). The addition of NT can effectively decrease methane production by rumen microbiome (Zhou et al., 2012; Newbold et al., 2014; Patra and Yu, 2014b), and its inhibition is explained by serving as a strong electron sink outcompeting CO_2_ for hydrogen (van Zijderveld et al., 2010; Asanuma et al., 2015) and by forming nitrite that is directly toxic to methanogens (Klüber and Conrad, 1998; Patra and Yu, 2014b). Instead of losing efficacy, NT decreased methane production to a greater extent over the course of the 18 days of *in vitro* incubation. This observation is consistent with that of a previous study (Zhou et al., 2012) and suggests that rumen microbiome cannot overcome NT inhibition of methane production. Methanogen abundance was not lowered by NT by day 2, but was decreased by day 18. The decreased methanogen abundance observed during the *in vitro* incubation may explain the greater inhibition of methane production by NT in the later days of the incubation. The inhibitory effect of NT on methane abundance was also observed in goats (Asanuma et al., 2015).

Saponins are toxic to rumen protozoa and thus may decrease methanogenesis through inhibition of protozoa-associated methanogens and their activities (Patra and Saxena, 2009). In the present study, protozoal abundance decreased in the QS-containing cultures except the ternary culture at day 2. However, no difference in protozoal abundance was seen among the cultures at day 10 or 18. It should be noted that protozoal abundance was lower at the two later days in all the treatments, indicating that the growth rate was lower than the dilution rate of the repeated transfer. The lack of QS inhibition to protozoa suggests that rumen protozoa can adapt to QS over time. Nonetheless, this study confirmed that GO, QS, and NT can inhibit methanogenesis through complementary modes of actions, additively/synergistically inhibiting methane production by rumen microbiome (Patra and Yu, 2015a).

Most of the methane inhibitors exert adverse effects on feed digestion and rumen fermentation at doses that achieve desirable mitigation of methane production. Garlic oil or its combination with NT or/and QS depressed feed degradability at the tested dose (0.25 mg/L) at days 10 and 18, though not at day 2, and the depressed degradability was accompanied with decreased abundances of fibrolytic bacterial population. In some *in vitro* studies (Pawar et al., 2014; Patra and Yu, 2015a), GO was evaluated only for a short period of time (24–48 h), missing the opportunity to determine its long-term effect. Future research is needed to determine a dose at which GO does not decrease feed degradation over long-term incubation. Saponin and NT at the tested doses did not affect digestibility of DM and NDF of the substrate over the course of the incubation. Lee et al. (2015) reported increased DM digestibility in beef heifers fed NT supplement. However, NT at high doses inhibited the growth of cellulolytic bacteria (Zhou et al., 2011, 2012) and *in vitro* DM digestibility (Marais et al., 1988). Saponin exerted variable effects on feed degradability depending upon concentrations, type, and feeding conditions (Patra and Saxena, 2009; Patra et al., 2012; Patra and Yu, 2013). These two anti-methane inhibitors may be used at farms to mitigate methane emission from ruminants. Of course, *in vivo* studies are needed to optimize the doses so that effective methane mitigation will be accompanied without decreasing feed digestion or fermentation.

### Effects on VFA and ammonia concentrations

The concentrations of total VFA were not altered by NT or QS, corroborating an *in vivo* study in which NT was fed at 2.2% of DM (Hulshof et al., 2012). However, in some studies concentration of total VFA was increased by saponins (Wang et al., 2009) and NT (Sar et al., 2004; Nolan et al., 2010; Patra and Yu, 2013). Garlic oil lowered the concentrations of total VFA, which was consistent with the depressed DM digestion. In an *in vivo* study, direct ruminal infusion of GO to goats at a dose of 0.8 g/day did not influence total VFA concentration or individual VFA proportions (Zhu et al., 2012). Garlic oil did not affect proportion of major VFAs, which was also observed in another study where propyl-propane thiosulfonate, a component of GO, was used at 0.2 g/L of rumen content (Martínez-Fernández et al., 2014). In contrast, GO (0.31 g/L, Busquet et al., 2005; 0.17 ml/L, Pawar et al., 2014) and propyl-propane thiosulfonate (0.05–0.10 g/L; Foskolos et al., 2015) increased the proportion of propionate and decreased that of acetate and branch-chained VFAs. Increased proportion of acetate and decreased proportion of propionate and butyrate in response to NT, as noted at day 2 in the present study, have been reported *in vitro* (Zhou et al., 2011; Patra and Yu, 2013) and *in vivo* (Sar et al., 2004, 2005; Hulshof et al., 2012). At day 10, the increased A:P ratio resulted from decreased concentration of propionate along with slightly increased concentration of acetate in all the NT-supplementing cultures Such changes were probably due to channeling of reducing equivalents toward reduction of NT by some rumen bacteria. Increased molar percentage of propionate and lowered A:P by QS, alone or in combination with GO and NT, at day 2 was accompanied with lowered protozoa abundance, but the responsible microbes for such a change remains to be determined. Butyrate percentage was lowered by QS, alone or in combinations with NT and/or GO. These characteristics of VFA profiles are generally observed in defaunated animals as the major end-products of fermentation of ruminal protozoa are acetate and butyrate (Williams and Coleman, 1997). Besides, inhibition of methanogenesis results in increased propionate concentration owing to re-channeling of excess hydrogen for propionate production. As expected and consistent with other studies (Sar et al., 2004; Hulshof et al., 2012; Patra and Yu, 2013), NT resulted in increased concentration of ammonia; however, when combined with GO and/or QS, the ammonia-promoting effect of NT was eliminated. Thus, GO and QS not only enhances methane mitigation by NT but also prevents elevated ammonia production that is inherent of feeding NT to ruminants. It remains to be determined if GO and QS can enhance nitrate-nitrogen utilization efficiency in the rumen.

### Effects on abundance and diversity of archaeal and bacterial communities

Garlic oil exerted the strongest inhibition to the growth of archaea, followed by NT, which was profound at the two later days, but not at day 2. However, QS had little effect on the abundances of archaea, which might be due to the low abundance of protozoa in the mixed cultures as discussed above. Corroborating with the finding of a previous study (Patra and Yu, 2015a), GO and NT together inhibited the growth of archaea to a greater extent than they did individually, suggesting that these two compounds have different complementary modes of action in decreasing methane production. In two previous *in vitro* studies, the abundances of *F. succinogenes* and *R. flavefaciens* were increased by QS (Patra et al., 2012; Patra and Yu, 2014b), while the abundances of these two cellulolytic bacterial species were not affected in this study at day 2. *R. albus* did not decrease in abundance at day 2 in the QS-containing cultures, but decreased at days 10 and 18. When used for long periods of time, QS may shift the populations of the three main cellulolytic bacterial species. The abundance of these cellulolytic bacterial species was not affected by NT, corroborating the findings of previous studies (Zhou et al., 2011, 2012; Patra and Yu, 2015a). However, NT at high doses was found to be toxic to rumen bacteria including cellulolytic bacteria (Marais et al., 1988; Zhou et al., 2012), probably due to the intermediary nitrite, not NT itself (Marais et al., 1988). Garlic oil inhibited the growth of *F. succinogenes*, but not the two cellulolytic ruminococci bacteria. Although combination of GO, QS, and NT at the tested doses was the most inhibitory to methane production, this treatment decreased feed digestibility and shifted fermentation profile. As discussed above, this is largely due to the adverse effect of GO. A dose-response study is needed to determine the optimal dose of GO when used in combination with QS and NT to achieve substantial methane reduction without adverse effects on feed digestion and fermentation.

The prominence or disappearance of DGGE bands suggests that the community composition of both archaea and bacteria was altered by the methane inhibitors. A microarray study also revealed that GO altered several rumen bacterial species *in vitro* (Patra and Yu, 2015b). Changes in microbial communities by NT, QS, and GO alone and in combinations had been reported earlier (Zhou et al., 2011; Patra and Yu, 2015a). The PCA plot of DGGE profiles demonstrated that the archaeal community in the cultures was altered to a greater extent by the methane inhibitors in combination than by them individually, which agrees with the effect of these inhibitors on the abundance of archaea. This study also showed that the cultures did not adapt to the ternary combination of methane inhibitors by day 18. Thus, this combination of inhibitors has potential to mitigate methane emission from ruminants.

## Conclusions

Combination of GO, NT, and QS additively decreased methane production by rumen microbiome, but the inhibition was alleviated to some extent over 18 days. Garlic oil at 0.25 g/L, either alone or in combination with NT and/or QS adversely affected feed degradability after 18 days of incubation. Thus, a lower GO dose needs to be determined before it can be combined with QS and NT to achieve effective decrease in methane production without adversely affecting feed degradability or rumen fermentation. This study suggests that GO, QS, and NT may be used together to mitigate methane emission from ruminant animal farms.

## Author contributions

AP planned, executed and written this manuscript. ZY planned and revised the manuscript.

### Conflict of interest statement

The authors declare that the research was conducted in the absence of any commercial or financial relationships that could be construed as a potential conflict of interest.
